# The Dilemmas and Opportunities of Co‐Creating Health Interventions to Fit Local Contexts: An Ethnographic Study on the Adaptation of Clinical Guidelines in Tanzania

**DOI:** 10.1111/hex.70073

**Published:** 2024-10-24

**Authors:** Haika Osaki, Morten Skovdal, Jane Brandt Sørensen, Nanna Maaløe, Natasha Housseine, Brenda Sequeira Dmello, Columba Mbekenga

**Affiliations:** ^1^ Department of Public Health University of Copenhagen Copenhagen Denmark; ^2^ School of Nursing and Midwifery (SONAM) Aga Khan University Dar es Salaam Tanzania; ^3^ Medical College Aga Khan University Dar es Salaam Tanzania; ^4^ Comprehensive Community Based Rehabilitation in Tanzania (CCBRT) Dar es Salaam Tanzania; ^5^ School of Nursing Kairuki University Dar es Salaam Tanzania

**Keywords:** childbirth care, clinical guidelines, co‐creation, ethnographic study, participatory research, Tanzania

## Abstract

**Introduction:**

Healthcare providers' role in co‐creating health interventions and implementation strategies has evolved significantly, and yet, there is little documentation of this from low‐resource settings. This study aims to share the dilemmas of engaging healthcare providers in co‐creating locally adapted clinical guidelines for maternity facilities in Dar es Salaam, Tanzania, and strategies used to address them.

**Methods:**

An ethnographic study explored the co‐creation of locally adapted clinical guidelines for childbirth care within five maternity facilities in Dar es Salaam. Participant observations were conducted during 11 co‐creation workshops. Six in‐depth interviews explored participant experiences. Data were analyzed using Attride‐Stirling's thematic network analysis framework.

**Results:**

The analysis revealed four themes representing dilemmas in the co‐creation process and strategies to improve co‐creation: (i) navigating diverse contexts: adapting a single set of guidelines to various, diverse facilities was challenging; this was addressed through engaging in dialogue and flexibility while adjusting care practices. (ii) Competing knowledge systems and sources: differing knowledge sources between researchers and healthcare providers challenged discussions on recommended practices. However, validating scientific recommendations with practical care experience in this context helped bridge this gap. (iii) Fostering meaningful participation: participation was time‐consuming for some. However, early stakeholder engagement and facility‐led participant selection facilitated the meaningful involvement of healthcare providers. (iv) Power imbalances: power dynamics influenced the co‐creation process; involving stakeholders in planning and co‐facilitating workshops helped mitigate these imbalances and encourage more equal participation.

**Conclusion:**

Navigating contextual variation, differences in knowledge systems, meaningful participation and power dynamics were key challenges in the co‐creation process. However, reflexivity, open and honest dialogue with stakeholders and early engagement enhanced the co‐creation process. Co‐creating locally adapted clinical guidelines with frontline healthcare workers and scientific experts is essential for feasibility and safety. Further research is needed to explore context specificity, decision‐making and the efficacy of co‐creation in low‐resource settings.

**Patient or Public Contribution:**

Healthcare providers and health managers from five maternity facilities who participated in the co‐creation process were actively involved in this study by providing their consent to be interviewed about their experiences of participation.

**Clinical Trial Registration:**

This study is a substudy within the PartoMa project. PartoMa is a registered clinical trial with the trial registration number NCT04685668. PartoMa's date of initial trial registration is 28 December 2020.

## 1 Introduction

The role of healthcare providers in developing health interventions and implementation strategies has significantly evolved in recent years. Healthcare providers are playing more central roles by co‐creating with researchers and other health professionals to strengthen the design and implementation of interventions [1, 2, 3]. The 1978 Alma Ata Declaration spearheaded participation in health planning and implementation by declaring health as a human right and emphasizing individual and community engagement to improve health outcomes [4, 5]. Although many African countries have a rich history of active participation by healthcare workers and community members in primary healthcare planning and service delivery, this level of engagement has not extended to the development of clinical guidelines [6]. In clinical guideline development, participation has predominantly been restricted to professionals and scientists, many of whom are based in the global North [7]. There is also a noticeable gap in research investigating strategies to engage healthcare providers, the actual end‐users of clinical guidelines, in the collaborative development of clinical guidelines [8]. Addressing this gap is essential for improving the adaptation and achievability of these guidelines.

As recommended by the World Health Organization (WHO) and other global developers, local adaptation of clinical guidelines is crucial for ensuring that guidelines are achievable within diverse contexts [9]. However, this is rarely done. In low‐resource settings, few guideline modifications are usually made and in maternal and newborn health particularly, significant gaps remain between guidelines and achievable best practices [10]. Consequently, healthcare providers are forced to make informal modifications such as reducing the frequency of foetal monitoring due to a high patient load [11]. This approach is unstructured, unsafe and unjust, highlighting the need for locally adapted, systematic and evidence‐based clinical guidelines to guide healthcare providers in low‐resource settings.

Through sharing ideas and collective decision‐making, co‐creation within clinical guideline adaptation has helped improve care in low‐ and middle‐income countries (LMICs). In Zanzibar, adapting international guidelines to suit national maternity services created a more achievable set of local guidelines for childbirth care [9, 12]. Similarly, in India, the adaptation of international guidelines at the national and state level was a strategy to improve and standardize clinical management in neonatology, mental health and other fields [13]. However, co‐creation processes in LMICs are also complex and resource‐intensive. Limited experience in co‐creation and volatile health systems in terms of funding, staffing, leadership and structure are common challenges [12, 14, 15]. Additionally, when contextual evidence is scarce or when global evidence does not align with local healthcare providers' experiences, it becomes difficult to approach decision‐making and stakeholder engagement during co‐creation [16, 17, 18].

With this background, we share our experiences of crafting co‐created clinical guidelines for childbirth care in Dar es Salaam, Tanzania, within the PartoMa project. The aim of our research study was to explore the dilemmas encountered by co‐creators during the development of locally adapted clinical guidelines for childbirth care and the strategies used to address them. We did this by exploring the perspectives and experiences of healthcare providers and other co‐creators involved in this process. We hope that our reflections on these dilemmas and strategies prove valuable as guidance for other researchers and healthcare professionals who plan to co‐create locally adapted clinical guidelines in the context of maternal health and beyond.

## 2 Methods

An ethnographic approach was adopted to study the PartoMa guideline co‐creation process and the participation of different co‐creators [19, 20]. Observations were conducted during all co‐creation workshops, and in‐depth interviews (IDIs) explored participant perspectives. Additional observations were conducted during two training sessions for healthcare workers and managers to support intervention implementation.

### 2.1 Co‐Producing Childbirth Care Guidelines: The PartoMa Project's Approach

Between June 2021 and December 2022, the PartoMa project engaged numerous maternal health stakeholders in co‐creating a set of locally adapted guidelines for childbirth care. These stakeholders included frontline health providers and healthcare managers from five maternity units with the highest number of births in Dar es Salaam, researchers, members from the regional and district health management teams and maternal health experts (see Table 1) [21].

**Table 1:** Details and roles of participants of the PartoMa co‐creation workshops.

Participants	Description and roles in the co‐creation
Maternal healthcare providers	*Description*: Obstetricians, doctors, nurse‐midwives and intern doctors from five major maternity facilities in Dar es Salaam, Tanzania
	*Role*: Shared experiences of providing care in the facilities and the existing care practices, reviewed the guidelines and provided suggestions for modification of the guidelines
Health managers	*Description*: Senior doctors and midwives, wards in‐charge and heads of department from the five study facilities
	*Role*: Contact persons in the facilities reviewed and gave feedback on the guideline content modification; co‐facilitated the co‐creation workshops
District and regional health management teams	*Description*: Health managers from the Dar es Salaam district and regional health management teams
	*Role*: Reviewed the guidelines and provided feedback for guidelines modifications to suit the context
Maternal health experts	*Description*: Local and international experts in midwifery, obstetrics and neonatology with extensive experience in low‐resource maternity care settings
	*Role*: Provided written peer reviews of the guidelines to ensure that modified guidelines were safe and aligned with the most recent scientific evidence
PartoMa researchers	*Description*: A multidisciplinary team of researchers
	*Role*: Primarily responsible for initiating, planning, organizing and documenting the co‐creation process. Responsible for gathering and sharing evidence to support the process and for documenting the modification of the guidelines.

Eleven co‐creation workshops were conducted, facilitated by one PartoMa researcher with a clinical background alongside one or two co‐facilitators. Co‐facilitators were obstetric gynaecologists and senior midwives from the study facilities. Including facilitators, workshops had between 9 and 14 participants. Five workshops were first conducted among separate groups of doctors (*n* = 2), nurse‐midwives (*n* = 2) and intern doctors (*n* = 1), who conducted a page‐by‐page review of the original PartoMa Zanzibar booklet and offered tailored suggestions to suit the study facilities. These homogeneous groups fostered open discussion among peers and mitigated the influence of professional hierarchy.

**Figure 1:**
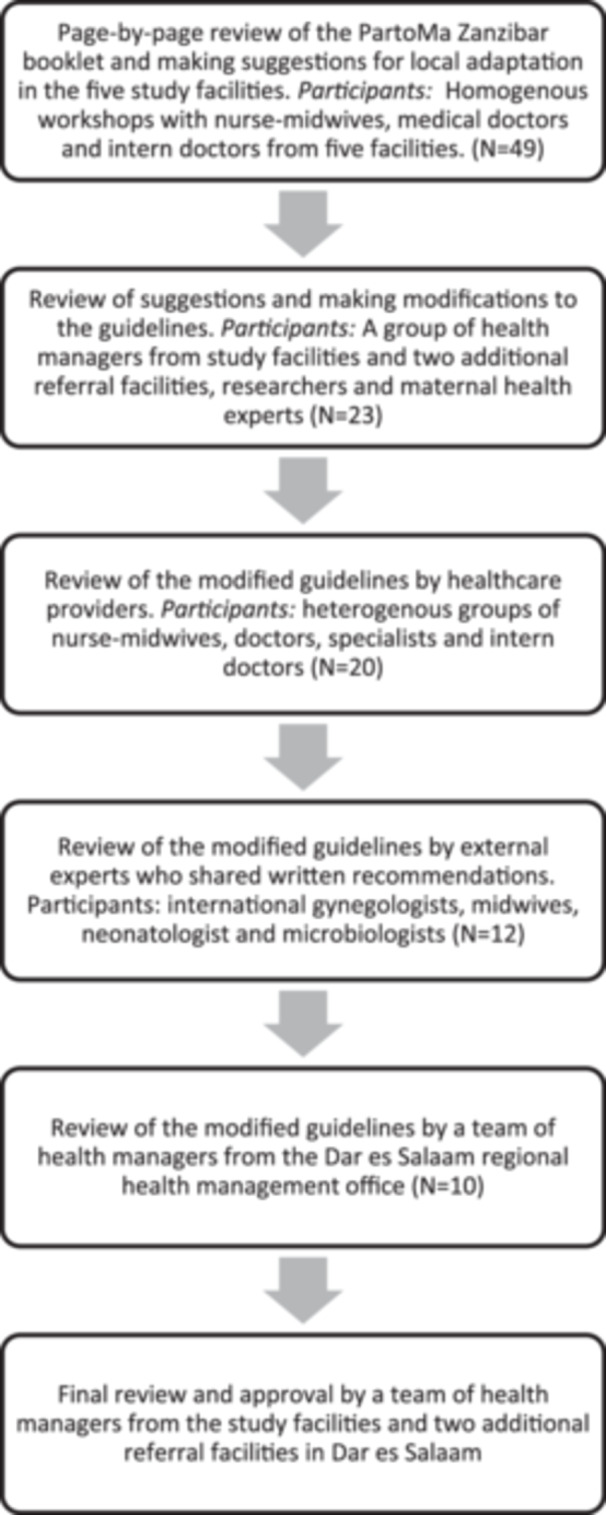
Flow of co‐creation workshops that took place in the PartoMa co‐creation process.

Afterwards, the tailored suggestions were reviewed, and modifications were documented during one workshop with health managers, researchers and maternal experts. Following the documentation of these modifications, multiple rounds of workshops and a peer review were conducted to improve and further refine the adapted guidelines. These included three workshops with mixed groups of healthcare providers (doctors, nurse‐midwives and intern doctors) and one workshop with a group of health managers from the regional health management office. Additionally, a group of maternal health experts (local and international specialists in midwifery, obstetrics and neonatology) also provided written peer reviews and recommendations of the modified guidelines via email. After multiple rounds of workshops and peer review, co‐creators finally arrived at an agreed final version of the adapted guidelines. The final version of the booklet was reviewed and approved during the final workshop in January 2022 by a team of health managers from the five health facilities, health managers from two national referral facilities and the Ministry of Health. Figure 1 shows the steps of the co‐creation process.

#### 2.1.1 Sampling and Study Participants

**Figure 2:**
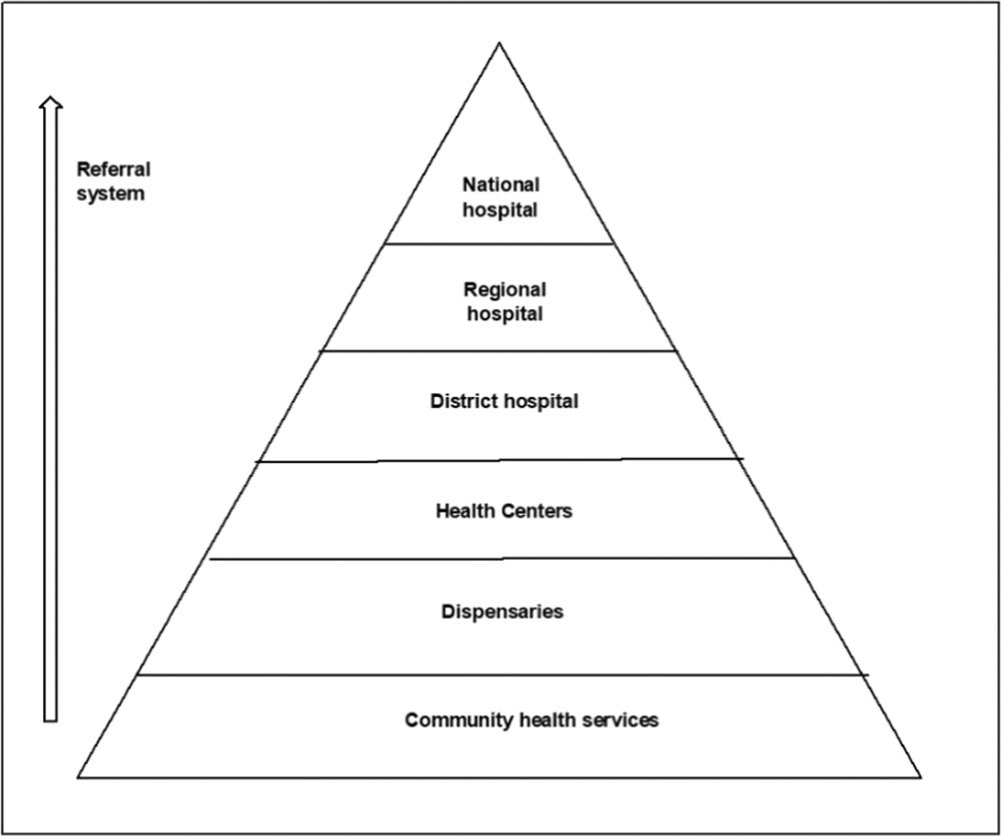
Levels of care in the Tanzania health system.

For convenience, health managers purposively selected maternity healthcare providers involved in labour care for women to participate in workshops. This aimed to minimize the disruption of tasks in the ward. Health providers were of different cadres and levels of seniority, including intern doctors, registrars, obstetricians, nurse‐midwives and midwifery specialists (collectively referred to as birth attendants). Health managers were heads of departments, specifically, obstetricians, midwifery specialists and wards in‐charge. Out of the five maternity units, three are referral hospitals, whereas two are health centres; all provide comprehensive emergency obstetric care that includes caesarean sections and blood transfusions (Figure 2 shows the levels of care in the Tanzanian health system). Purposive sampling was used to select workshop participants to participate in IDIs. A list of names and contact details of potential participants was compiled at the end of each workshop. Participants were selected based on their level of engagement during workshops and their willingness to take part in an interview after being approached. To ensure diversity in shared experiences and perspectives, participants with various roles from all five facilities, including doctors, nurse‐midwives and specialists, were included.

#### 2.1.2 Data Collection

##### 2.1.2.1 Participant Observation

Participant observation was conducted between June and November 2021 by H.O., a social scientist and experienced qualitative researcher within the PartoMa research team. At the start of every workshop, H.O. introduced herself as a member of the research team and informed participants of her role in observing and note‐taking during workshops. Observations focused on workshop activities, discussions and interactions between participants and facilitators. Informal conversations with participants took place during breaks. Detailed notes were taken during each workshop, totalling 69 pages of single‐spaced text. As the researcher conducting the study is not a clinician, her contribution during workshops was limited to asking clarifying questions and photographing workshop activities for documentation. Additionally, H.O. observed two PartoMa training sessions for facilitators to prepare for subsequent seminars for the implementation of the co‐created guidelines in the facilities. The training, facilitated by two research team members, involved obstetricians, nurse‐midwives and medical doctors from the study facilities. It included familiarization with the co‐created guidelines, facilitation practice and reflection on prior seminar sessions. H.O. was interested in concerns raised and challenges faced during initial seminar sessions or regarding guideline implementation.

##### 2.1.2.2 IDIs

To further explore the co‐creation process, seven IDIs were conducted: six with healthcare providers and one with the lead researcher and facilitator (N.H.). Participants were contacted and invited to schedule an interview at their convenience. To avoid disrupting care responsibilities, all interviews with healthcare providers were conducted during breaks or after shifts in a private room at their health facility. A semi‐structured interview guide was used (see Supporting Information Material S1). Participants were asked about their opinions of the workshop structure, their experience as participants and reflections on what went well and what could be improved. Interviews were conducted in Kiswahili, English or both languages depending on participant preference and audio‐recorded upon participant consent. Interviews ranged between 30 min and 2 h. All interviews were conducted by HO, who is fluent in Kiswahili and English. Audio recordings were transcribed verbatim. Table 2 presents the details of the interview participants.

**Table 2:** Details of in‐depth interview participants.

ID	Position	Sex	# of workshops attended	Place of employment
1	Obstetrician	M	2	Health centre
2	Medical doctor	F	2	Regional referral hospital
3	Intern doctor 1	F	2	Regional referral hospital
4	Intern doctor 2	F	2	Regional referral hospital
5	Senior midwife	F	3	Regional referral hospital
6	Nurse midwife	M	2	Regional referral hospital
7	PartoMa Researcher	F	9	PartoMa project

### 2.2 Analysis

The data analysis process began during data collection and took place as a continuous and iterative process. During data collection, the authors met regularly to discuss emerging findings from observations and interviews. After data collection was complete, the data were subjected to formal analysis to systematically explore the narratives surrounding the experiences and perspectives of the co‐creation process. All data were subsequently imported into Nvivo 12 software [22] for coding and thematic analysis, following the steps for thematic network analysis as outlined by Attride‐Stirling [23].

First, observation notes and interview transcripts were reviewed and discussed by H.O., M.S., J.B.S. and C.M. to gain a deeper understanding of the data. Second, codes were initially formulated on the basis of reoccurring descriptive themes in the data. Third, after revising patterns, codes were then grouped into basic themes, some of which highlighted the various challenges encountered during the co‐creation process and others illustrated strategies to address these challenges. The fourth step involved grouping basic themes into four overarching and more interpretative organizing themes, each illustrating a core dilemma, and the strategies used to address the specific dilemma. Finally, the four organizing themes were presented, summarizing the main argument for this manuscript, which is the global theme: Dilemmas and opportunities of co‐creating locally adapted clinical guidelines. The global theme and the organizing themes serve as the structure for our findings section. Themes and codes were presented and discussed between co‐authors for further clarity and depth. All themes were agreed upon by the co‐authors before being finalized. Table 3 illustrates our thematic network and how codes and themes relate to each other.

**Table 3:** Thematic network (from codes to global theme).

Codes	Basic themes	Organizing themes	Global theme
Facilities have many patients seeking care. Some suggested practices will depend on whether drugs are available. Misoprostol is not used by one facility due to no staff for monitoring. Most facilities have no space to allow birth companionship. Birth companions are allowed for young mothers and sick women. Facilities need more equipment to achieve some guidelines. It is not possible for us to monitor foetal heart every hour. Only special cases are monitored more frequently. Some wards do not have electronic monitors. One‐hour intervals for monitoring should remain the standard for all. Monitoring can be adjusted at the facility level. Contractions are not measured, too time‐consuming. We do not provide vitamin K doses in our facility.	1. A high burden of care, differences in resources. 2. Differences in staff number and training. 3. Variation in space and facilities in the hospitals. 4. Some facilities are at a higher level compared with others. 5. Staff acknowledge that care practices vary in the facilities. 6. Adapting monitoring in all facilities is challenging.	Co‐creating across diverse contexts	Dilemmas and opportuniti of co‐creating locally adapted clinical guidelines
Guidelines in the facility are present, but not used. Guidelines at one facility are locked in a cabinet. Guidelines in our facility are the experiences of the nurses. Guidelines are not available; we consult the senior. Guidelines can be conflicting, for example, partograph opening. From experience, the partograph is opened at 4 cm. Trial of scar is safer than surgery. From experience, trial of scar is dangerous. Decision‐making on trial of scar depends on resource availability. Oxytocin use should be reduced. Oxytocin helps speed‐up labour. Oxytocin reduces congestion. Reducing oxytocin could increase congestion. We managed to reduce oxytocin use in the ward. Reducing oxytocin helped reduce caesarean section and foetal distress.	1. Clinical experience is more highly regarded than evidence. 2. Experience is used to settle conflicting guideline recommendations. 3. Care decision‐making relies on resource availability. 4. Changing care practices requires an increase in facility resources. 5. It is possible to adjust some practices without major structural changes, for example, oxytocin use.	Competing knowledge systems and sources	
Co‐creation process was hard to plan. There were delays in the planning process. We had a slow start. Planning meetings took time to organize. Facility management selected workshop participants. Participants selected according to facility convenience. Health managers played part as workshop co‐facilitators. Co‐facilitators openly answered questions. Co‐facilitators shared similar experiences of working in the facility. Co‐facilitators encouraged us to speak. Co‐facilitation was time‐ and energy‐consuming. Co‐facilitation required use of experience, skills and knowledge. Workshop co‐facilitation is work. Compensation helped support workshop attendance. Compensation supports motivation for attendance. We appreciate compensation for participation.	1. Collaborators were involved in the planning process. 2. Planning for co‐creation was challenging and time‐consuming. 3. Co‐facilitators were important for supporting workshops. 4. Co‐facilitation was time‐ and labour‐consuming 5. Compensation is important for participation. 6. Are co‐facilitators adequately compensated for their work?	Fostering meaningful participation while avoiding disruption	
Co‐creators have different positions, influence. Research team decided on the research problem. Research team chose the design of the intervention. Research team controls and distributes resources for co‐creation. Research team makes modifications to the guidebook. Guidebook reviewed over multiple rounds. Research team shares scientific evidence during workshops. Healthcare workers know accepted clinical practices. Healthcare workers are final decision‐makers for care. Healthcare workers are implementers of the guidelines. Health management selected workshop participants. Health managers played part as workshop co‐facilitators. We used soft negotiation during discussions. We felt that we could openly share our challenges. Our discussions were judgement‐free.	1. Research team is in charge of initiating the co‐creation process. 2. Research team manages resources for co‐creation. 3. Healthcare providers in charge of implementing care. 4. Open dialogue helped facilitate discussions. 5. Soft negotiation and empathy supported open discussion. 6. Shared facilitation supported the co‐creation process. 7. Planning involved the co‐creators.	Imbalance of power among co‐creators	

### 2.3 Ethics

We obtained ethical clearance from the National Institute for Medical Research in Tanzania (NIMR) (*NIMR/HQ/R.8a/Vol. IX/3324, NIMR/HQ/R.8c/Vol. I/1679, NIMR/HQ/R.8c/Vol. I/926)* and a research permit from the Tanzania Commission for Science and Technology (COSTECH). Study participants were informed that the study focuses on exploring the process of co‐creating guidelines, emphasizing that their participation was voluntary and they could withdraw at any point. All participants consented to be observed during workshops and provided written informed consent before participating in discussions or interviews.

## 3 Findings

During co‐creation workshops, four key dilemmas arose, challenging the process: (i) co‐creating across diverse contexts, (ii) competing knowledge systems and sources between co‐creators, (iii) fostering meaningful participation and (iv) imbalance of power among co‐creators. We outline these dilemmas and strategies in Table 4.

### 3.1 Co‐Creating Across Diverse Contexts

Adapting one set of guidelines across five facilities was a challenge. Despite a shared high burden of care, facilities differed in their ward space, ratio of provider to woman, the level of care of the facility (e.g., secondary referral hospital vs. primary health centre providing comprehensive obstetric care) and number of healthcare providers. Healthcare providers acknowledged and considered this variation during co‐adaptation:

I realized that we do things differently in the facilities … we are not the same. (IDI, Doctor)

The variability in context raised questions about the feasibility of co‐adapting one set of guidelines across multiple facilities. To address this dilemma, co‐creators, that is, birth attendants, managers and researchers, negotiated a standard of care for specific practices. However, they also acknowledged that some practices may require further adaptation to suit specific hospital contexts and the condition of very ill women. For example, they agreed to recommend foetal heart monitoring at least hourly instead of every 15–30 min as recommended by international guidelines [24]. They also recognized that monitoring frequency would likely be lower when the ratio of provider‐to‐woman was high and would vary depending on the type of monitor available – a Pinard stethoscope (lower frequency) or a hand‐held Doppler (higher frequency).

Some healthcare providers added that monitoring was crucial, and they often had to prioritize close monitoring of very ill women even when the ward was busy. Although the use of a birth companion for women was generally agreed upon, it was difficult to achieve due to insufficient ward space, leading to congestion in the facilities. However, healthcare providers also acknowledged that sometimes, companions were allowed for very vulnerable women such as underage mothers and very ill women needing close monitoring. To address this dilemma, co‐creators agreed that birth companionship would be allowed for vulnerable women, offering hope that this practice would be achievable for all women in the future.

It was very tricky to keep [birth companionship] in the booklet when we agreed it was difficult to do. But we kept it in because it is more for the future … [we hope] that one day, in this setting, a woman can give birth with her sister, her mother or her husband present to support her … and we see that in some of the facilities here in Dar es Salaam, it is already being practiced. So, it is something that is difficult to do now, but it is something to aim for in the future. I know the Ministry is also trying to change these conditions so that is also a sense of hope…. (Informal conversation, Researcher, and facilitator)

To address the variability across facilities, flexibility was embedded into the guidelines, encouraging healthcare providers to make additional adaptations. This recognized the need for achievability in the adapted guidelines and variability within contexts while also upholding high standards of care.

### 3.2 Competing Knowledge Systems and Sources Between Co‐Creators

During discussions, the research team drew on scientific evidence prescribing ideal clinical practices, for example, recommended practices by governing bodies (WHO and the Tanzanian Ministry of Health) and the latest scientific evidence. Healthcare providers, on the other hand, primarily tapped into their collective knowledge of practicing care within the facilities. These different sources of knowledge resulted in a clash between scientific evidence and practical experience, which became evident in several ways.

Although healthcare providers recognized the importance of converting evidence into practice, they found existing, international guidelines inaccessible and unhelpful for their contexts. An intern doctor shared the following when asked about the use of existing guidelines in the facilities:

We do not use guidelines. For us, guidelines are the experiences of the midwives … the booklet is not easily accessible … we learn from experience. (Intern doctor, Observation notes, 21 June 2021)

Others mentioned how guidelines were collecting dust or locked away. This favouring of experiential knowledge over specific guidelines raised questions about the usefulness of the PartoMa guidelines if they fail to resonate with clinical reality. This emphasized the importance of adapting achievable guidelines, increasing the likelihood of their active use while balancing clinical safety.

Additionally, the contradictory and hierarchical nature of clinical guidance from different authorities was a source of contention. For instance, the Tanzanian Ministry of Health and the WHO have differing guidelines concerning when to start charting the partograph while monitoring childbirth (3 and 5 cm dilatation, respectively). Due to delays in updating the Ministry's guidelines, nurse‐midwives shared that they experimented with both recommendations and settled on starting the partograph at 4 cm, as it reduced the chances of prolonged labour based on their observations of patient outcomes.

Although health managers were aware of recent scientific evidence and clinical guidelines, they remained sceptical of new evidence challenging existing care practices within their facilities. During a workshop, the research team shared recent evidence challenging the assumption that labour progresses at 1 cm per hour cervical dilatation. This evidence recommended allowing more time for women with slower labour to progress before using oxytocin to quicken delivery. Health managers doubted this evidence, expressing concerns that this change would challenge the important role of oxytocin in the facilities.

The challenge is that they said oxytocin should not be used as much but for us, oxytocin is what helps us speed up delivery … it helps shorten the woman's labor which is better than leaving her alone when you depend on contractions … when we were told that it is supposed to be used at a different time or not used at all, that really confused us…. (IDI, Nurse‐midwife and Ward in‐charge)

The confusion caused by this new evidence was not only limited to the changes in oxytocin use but also the required structural changes. A specialist shared that the facility management had no control over the necessary structural changes to support this change.

If it is applicable here, it would also require strengthening other areas around the labor process … like the setting, the number of beds, you would have to increase skills, and we would need enough facilities like the theatre. Under the current circumstances, I don't think that's possible … it's more of a political and financial issue, we do not make these decisions … they are made in higher‐ranking organizations…. (IDI, Specialist and Head of department)

Although healthcare providers acknowledged the dangers of oxytocin use, they were sceptical about changing the practice. However, shared experiences from healthcare providers who attempted to reduce oxytocin were highly valued among participants. During a training seminar for implementation facilitators, a doctor shared his attempts to reduce oxytocin use in his facility due to the absence of a functional theatre, while staff also had to manage increased congestion in the ward. This was successfully achieved while also reducing the Caesarean section rate and negative birth outcomes for women and babies. This intrigued and reassured other healthcare providers that change was possible, and congestion could be manageable. Healthcare providers, however, only entertained the idea of reducing oxytocin use when the scientific evidence was corroborated with experiential evidence, further demonstrating that frontline practitioners' experiences outweighed evidence‐based recommendations.

### 3.3 Fostering Meaningful Participation

The PartoMa co‐creation process primarily depended on the participation of key stakeholders and permission from national, regional and district governing bodies. Planning the co‐creation was a challenge, as there were delays in receiving responses from the health facilities and the regional health management team. For the research team, this was disconcerting, as it created some uncertainty about the health facilities' interest and engagement and how meaningful participation from collaborators could be achieved.

[The co‐creation] was quite hard to plan … I was always uncertain of the engagement from the hospitals. Sometimes we would write letters and wonder if the letters have been processed … have they selected participants, will they come? We also had a very slow start … it took a long time to get meetings organized with [the regional health team] … there were many delays at the beginning. (Informal conversation, Researcher, and facilitator)

Despite initial concerns, workshops had strong attendance with good collaboration among co‐creators. As co‐facilitators, health managers nurtured and encouraged participation strengthening overall project ownership. One participant shared:

I think they were helpful to us, I thought [the co‐facilitator] was also part of the research team … they helped encourage us to speak, it was very nice … they were a big help to us because whenever we had a question, they were able to respond openly so that everyone understood, they helped us a lot. (IDI, Nurse‐midwife, and health manager)

However, meaningful participation from co‐facilitators came at a cost. Co‐facilitators dedicated substantial time, effort and expertise to the process while maintaining leadership and care responsibilities in the facility. Although the co‐creation intended to strengthen clinical care, the dilemma for the research team was that this required healthcare providers to spend time away from their care responsibilities. To mitigate this, facilities self‐selected participants and strategically limited the number of participants to reduce shortages.

Furthermore, although co‐facilitators were offered compensation like other participants, they assumed more responsibilities during the process, raising an ethical dilemma. Initially, the research team did not plan to compensate participants, as healthcare providers' engagement in co‐creation was a means to improve clinical practice. However, during the planning and co‐creation phase, the significant effort of co‐facilitators challenged this assumption. The lead researcher of the co‐creation process reflected on this dilemma:

Originally, we said that co‐facilitators do not need to get compensated [because] participation was voluntary, and no compensation was provided during the Zanzibar pilot phase. But then I realized the co‐facilitators were working … they did a lot of work. It did not make sense to just give them an allowance…. (Informal conversation, Researcher, and facilitator)

Although this dilemma was acknowledged, additional compensation could not be provided due to budget constraints. This experience challenged the budgeting process for co‐creation, emphasizing the need to include more compensation for co‐creation activities in the future. This is especially evident for participants with extensive responsibilities such as co‐facilitators. Additionally, giving space to health facilities to independently select participants mitigated the anticipated consequences of engaging frontline healthcare providers.

### 3.4 Imbalance of Power Among Co‐Creators

During co‐creation, emphasis was placed on ensuring that all participants had an equal opportunity to voice their opinions and contribute meaningfully. Yet, in realizing this equality, several challenges emerged due to the diverse power dynamics and varying levels of influence among participants.

As initiators of the co‐creation process, the research team determined the research problem, and intervention design, and controlled funding and allocated resources. Although healthcare providers were invited to participate in the co‐creation, they did not possess control of funding but received a transport allowance for their participation. Researchers and external experts also had better access to scientific knowledge, the basis of clinical guidelines, whereas healthcare providers had a greater understanding of the realities of providing care in health facilities and what was achievable. The research team was solely responsible for translating feedback from workshops into written modifications of the guidelines in the booklet, granting them authority over the interpretation and articulation of the guidelines. However, the research team's authority was confined to the collaborative process and did not extend to guideline implementation at the facility level. Healthcare providers, on the other hand, oversaw the implementation of the adapted guidelines through their decision‐making regarding care.

To support a balanced and collaborative environment among co‐creators, efforts were made to share power. This involved involving representatives from the health facilities during the initial planning phase of the co‐creation. This allowed for the health facilities to participate in deciding how, when and where the co‐creation would take place. It also promoted a fair allocation of roles and responsibilities among collaborators, where workshop facilitation was not solely assigned to the research team but also to health managers from the facilities.

When we were planning the co‐creation, I suggested that we pair our facilitator with somebody local who can also play a co‐leading role in facilitating the workshops. I did not really expect that they would be that involved but I am very happy that they [took charge] in facilitating the discussions…. (Informal conversation, Researcher, and facilitator)

Health managers demonstrated strong leadership in co‐facilitation, allowing the research team to focus on organizing workshop activities and documenting agreed adaptations to the guidelines.

Recognizing that healthcare providers ultimately held responsibility for implementing guidelines motivated continuous and positive dialogue regarding clinical practices. This positive dialogue was facilitated through a strategy of ‘soft negotiation’ that refrained from critiquing or imposing judgement toward healthcare providers' deeply rooted care practices that were not meeting international recommendations. For example, instead of imposing judgement regarding the use of slow‐acting drugs, the use of rapid‐acting drugs was encouraged (when possible) while acknowledging the associated higher costs. Soft negotiation also meant more focus on finding common ground between evidence and achievable practice. This was important for building trust and nurturing honesty about clinical realities. Healthcare workers' honesty about their clinical environment and practices thus communicated their needs and concerns regarding care provision.

Additionally, to ensure that the research team adequately addressed workshop recommendations, co‐creators engaged in continuous review of the modified guidelines to ensure that modifications were documented and interpreted in line with the agreements reached during workshops. During review, healthcare providers freely pointed out any guidelines that had not been modified according to previous agreements, allowing additional adjustments where necessary.

**Table 4:** A summary of dilemmas faced during co‐creation and key strategies for supporting active and more equal co‐creation during clinical guideline adaptation.

Dilemma in co‐creation	Key strategies for supporting co‐creation
Co‐creating across diverse contexts	Encouraging flexibility to accommodate contextual variation. Open and honest communication about what is and is not achievable within different contexts.
Competing knowledge systems and sources between co‐creators	Acknowledging the hierarchy of value of evidence. Validating scientific recommendations by comparing them with shared experiential evidence from real‐life experiences within relevant, local contexts.
Fostering meaningful engagement while avoiding disruption	Close engagement of a few key actors (health managers) who can influence change and decision‐making. Providing compensation to support participation in co‐creation activities. Shared facilitation of co‐creation discussions among co‐creators.
Imbalance of power among co‐creators	Early engagement of all co‐creators in the co‐creation planning process. Shared facilitation of co‐creation discussions among co‐creators. Engaging in positive dialogue using soft negotiation.

## 4 Discussion

In this paper, we share the dilemmas of co‐creating locally adapted, clinical guidelines to support childbirth care in five maternity facilities in Tanzania. The co‐creation process served as a crucial coping mechanism, enabling stakeholders to engage in collective decision‐making regarding evidence‐based, achievable clinical guidelines in a resource‐limited setting. Although valuable, the co‐creation process was time‐ and resource‐intensive and presented some dilemmas that promoted adaptability to the study settings and nurturing inclusivity among participants. As we unfolded, dilemmas encountered involved complexities in navigating diverse clinical contexts and differing perceptions of evidence between researchers and healthcare providers. Co‐creation also presented challenges in the meaningful engagement of healthcare providers and a need for balancing power dynamics among co‐creators during the process. These dilemmas were resolved by numerous strategies that promoted nonjudgemental dialogue during the co‐creation workshops and flexibility of the adapted guidelines to accommodate local variations. Additionally, encouraging inclusivity of healthcare providers in the planning and facilitation of co‐creation workshops fostered more equal power dynamics and meaningful participation among healthcare providers. These dilemmas are not limited to co‐creation within clinical guidelines but are also relevant to other health interventions.

Co‐creating clinical guidelines across various contexts requires acknowledgement of variations and an understanding of contextual factors. Variations in staffing, clinical materials, hospital organization, leadership and patient numbers over time influence local adaptation of guidelines in low‐resource settings. In recent years, health facilities in urban areas in African countries have failed to catch up with the growing population [25]. Additionally, McNab and colleagues [26] have criticized the current mental model for structuring maternal and neonatal interventions in urban contexts, claiming that it fails to recognize the unique and evolving contextual factors in rapidly growing African cities. These factors alongside shocks such as the recent COVID‐19 pandemic have weakened maternal and neonatal health in urban areas, leading to an urban disadvantage in maternal health [27]. To address this, co‐adapted guidelines require flexibility that enables healthcare providers to adjust practices according to what is achievable under existing conditions. As argued by Redman et al. [28], open communication with healthcare providers during co‐creation is crucial in effectively incorporating flexibility into guidelines during modification. As our findings suggest, embedding flexibility is beneficial when there are diverse contexts for adaptation; however, it also carries the risk of straying from scientific evidence. Therefore, it is essential to conduct a thorough guideline review, involving experts familiar with the evidence, and to document the rationale for specific modifications [13]. This approach demonstrates that guideline adaptation is a continuous process that should occur over time.

The clashes between scientific evidence and healthcare providers' experiential knowledge illustrate the gap between global evidence and the realities of clinical practices in unique contexts. To establish common ground, an honest dialogue is necessary to comprehend the different perceptions of evidence and, if possible, agree on feasible and acceptable practices within local contexts [29].

In this study, healthcare providers were hesitant to accept evidence and recommendations on reducing oxytocin during labour. Though literature is scarce, English and colleagues share similar experiences from their work where healthcare providers were reluctant to immediately accept some guideline recommendations [30]. This rejection of guideline recommendations highlighted how some clinical practices are administered to cope with the challenging clinical environment. A notable example is Kujabi et al.'s [31] study revealing the use of oxytocin in an urban maternity unit as a strategy to manage overcrowding.

Our findings highlight the implications of scientific evidence and subsequent guideline recommendations on the broader healthcare system, beyond individual clinical practices among providers. To navigate these complexities, researchers must acknowledge the hierarchy of value of evidence among co‐creators and the determinants of this hierarchy. Researchers and experts must also advocate for more conducive healthcare systems in low‐resource settings and recommend the best possible practices to combat inequalities and healthcare needs [10].

Recognizing power dynamics is crucial during co‐creation, as researchers and experts often hold more power than community members or healthcare providers [32]. However, in our context, although researchers and experts managed resources for conducting the co‐creation, healthcare providers held authority during guideline implementation post‐creation. Therefore, practices such as involving healthcare facilities during co‐creation planning, engaging healthcare providers as co‐facilitators, continuous review of the modified guidelines after initial workshops and soft negotiation promoted a balance of power among collaborators [14]. Similarly, Worsley et al. [33] show that positive communication and support among collaborators facilitate honest discussions and create a sense of equality among stakeholders. Further, the inclusion of local researchers and non‐governmental organizations with experience on research topics and setting is important for balancing power dynamics during co‐creation [34].

Studies highlight meaningful engagement as crucial for successful co‐creation with stakeholders. However, this requires investment of time, skills and expertise and could increase staff shortages in low‐resource facilities by keeping frontline healthcare providers away from their clinical duties [34, 35]. Yet, it is possible to engage healthcare providers in an acceptable way that minimizes disruption within health facilities. This can be achieved by focused engagement of experienced individuals who can influence change. However, the engagement of a select few key persons can also raise questions regarding inclusivity in co‐creation, highlighting the importance of considering whose knowledge and experiences are valued [36]. Utilizing existing systems and strategies within health facilities may be more feasible than introducing new ones. Further, restructuring research budgets to support the complex stakeholder engagement required for long‐term partnerships rather than one‐off co‐creation projects is essential [28]. To facilitate this effectively, there is a need to narrow disparities between global guidelines and local realities in LMICs [10].

### 4.1 Strengths and Limitations

This study presents original empirical data delving into the complexities of co‐creating adapted clinical guidelines for childbirth care in an LMIC. Through first‐hand experiences and perspectives of diverse co‐creators, we explore the potentials and challenges of co‐creation, offering tangible solutions to dilemmas faced. Our findings and analysis address a gap in literature on co‐creation, specifically in the context of clinical guideline adaptation within LMICs, providing guidance for similar endeavours. The qualitative methodology that we use draws from diverse data sources, offering a multidimensional perspective.

One limitation of this study is that our findings may be subject to potential bias due to the qualitative methodology used, which heavily relies on the researcher's interpretation and perspective during the data collection and analysis. To address this, data analysis commenced during data collection phase, where the lead researcher engaged in continuous discussion about the findings with a multidisciplinary team of local and international researchers. The process allowed a more diverse interpretation of the findings. Additionally, our study mainly focused on the experiences of healthcare providers and researchers. We did not conduct interviews with maternal health experts to explore their perspectives; therefore, they may have had different views to share regarding the process.

### 4.2 Implications for Clinical Practice and Future Research

Local adaptation is crucial for the feasibility of clinical guidelines. Frontline healthcare workers and scientific experts must co‐create guidelines to ensure safety and achievability. Further research is needed to examine the implications of context specificity on the scalability of adapted guidelines in low‐resource settings. Additionally, more research is needed to explore how co‐creation processes take place in low‐resource settings and how decision‐making takes place among co‐creators to ensure equitable and effective participation of stakeholders. To assess the efficacy of co‐creation as a method of engagement, more studies on the implementation and evaluation of co‐created interventions are needed.

## 5 Conclusions

Ultimately, our findings demonstrate the dilemmas of engaging in the co‐creation of clinical guidelines in a low‐resource setting and strategies used to navigate these challenges. Although contextual variation, differences in knowledge systems among co‐creators and navigating power dynamics and engagement challenged the process, we found that integrating flexibility during co‐creation and fostering open, honest dialogue about achievable practices were effective in addressing contextual differences. Furthermore, validating scientific knowledge with shared practical experiences played a role in establishing trust during knowledge exchange. The close involvement of a select few influential actors facilitated meaningful participation along with offering compensation for engagement in co‐creation activities. To ensure a balanced distribution of power among co‐creators, we found that early engagement in the planning of co‐creation activities, combined with shared facilitation and positive dialogue, supported more equitable participation among co‐creators. Although the co‐creation of context‐specific clinical guidelines represents a step toward improving childbirth care and outcomes in low‐resource settings, enhancing healthcare systems in urbanizing areas in LMICs remains imperative. This requires adequate healthcare providers, training and medical materials to address women's childbirth needs.

## Author Contributions

**Haika Osaki:** conceptualization, writing–original draft, writing–review and editing, formal analysis, data curation, investigation, software, methodology. **Morten Skovdal:** conceptualization, funding acquisition, methodology, writing–review and editing, supervision, formal analysis. **Jane Brandt Sørensen:** conceptualization, funding acquisition, writing–review and editing, supervision, methodology, formal analysis. **Nanna Maaløe:** conceptualization, funding acquisition, writing–review and editing, supervision, methodology. **Natasha Housseine:** conceptualization, funding acquisition, writing–review and editing. **Brenda Sequeira Dmello:** writing–review and editing. **Columba Mbekenga:** conceptualization, funding acquisition, writing–review and editing, supervision, methodology, formal analysis.

## Ethics Statement

We obtained ethical clearance from the National Institute for Medical Research (NIMR) in Tanzania (*NIMR/HQ/R.8a/Vol. IX/3324, NIMR/HQ/R.8c/Vol. I/1679, NIMR/HQ/R.8c/Vol. I/926*) and a research permit from the Tanzania Commission of Science and Technology (COSTECH). All workshop and interview participants provided written informed consent before participating in focus groups or interviews.

## Conflicts of Interest

The authors declare no conflicts of interest.

## Supporting information

Supporting information.

## Data Availability

The data sets generated and analyzed during the current study are not publicly available because study participants did not consent to share their information publicly. Data summaries are available from the corresponding author on reasonable request.
