# Supplementary material for: The Dilemmas and Opportunities of Co‐Creating Health Interventions to Fit Local Contexts: An Ethnographic Study on the Adaptation of Clinical Guidelines in Tanzania
Source: Health Expect. 2024 Oct 24;27(5):e70073. doi: 10.1111/hex.70073 (PMC11500208; doi:10.1111/hex.70073)
Supplement: Supplementary file 1 — Supporting information. [file HEX-27-e70073-s001.docx]

*Introductory note*

I am a student/researcher and part of a Tanzanian and international team that looks into health care workers’ and managers’ experiences with working at the maternity wards at several hospitals in Dar es Salaam Region. Thank you for taking the time to talk to me about your participation in the PartoMa workshops that took place between June 14^th^ to June 24^th^ 2021.

You have been asked to take part in this interview because you were one of the participants in the PartoMa co-creation workshops who participated in sharing feedback on the PartoMa guideline booklet. Your responses and perspectives will be kept strictly confidential. Your name will not appear anywhere. If you are uncomfortable participating in this interview, you are free to let me know and we will stop the interview. If you want to take a break and continue at another time, please let me know. Your experiences are very important to us. Keep in mind that we are interested in both negative and positive comments. We would like to learn from your sincere opinions and experiences, so please do not fear to give your honest answers. Your experiences are very important to us.

There are no right or wrong things to say – we are only interested in your experiences and perspectives. The interview will take approximately one hour. Can I have your permission to participate in this interview? [If yes] Can I have your permission to record the discussion? In this way, I do not have to write down all your comments while we talk. All recordings will be transcribed and then deleted. Do you have any questions before we start the focus group discussion?

***Turn on recorder.***

***--------------------------------------------------------------------------------------------------------------------------***

*Mimi ni mwanafunzi/mtafiti na sehemu ya timu ya Tanzania na ya kimataifa inayoangalia uzoefu wawafanyakazi wa afya na mameneja wafanyao kazi katika wodi za uzazi katika hospitali kadhaa za Mkoa wa Dar es Salaam. Asante kwa kupata muda wa kuongea na mimi kuhusu ushiriki wako katika warsha (workshop) za kufanya maboresho ya muongozo wa utoaji huduma ya uzazi uliotengenezwa na PartoMa. Warsha hizi zilifanyika kati ya tarehe 14 Juni na tarehe 24 Juni mwaka 2021.*

*Umeombwa kushiriki kwenye usaili huu kwa sababu ulikua mmoja wa washiriki aliotoa mrejesho juu ya kitabu cha muongozo wa utoaji huduma ya afya cha PartoMa katika warsha (workshops) za PartoMa. Majibu yako yatakuwa siri kabisa. Jina lako halitaonekana popote. Kama haujisikii kushuriki kwenye majidiliano haya, uko huru kuniambia na tutasitisha usaili. Kama ungependa kupumzika na kuendelea muda mwingine, tafadhali nijulishe na tutapanga hivyo. Uzoefu wako ni muhimu sana kwetu. Kumbuka kwamba tungependa kupata mawazo hasi na chanya (mabaya na mazuri) na tungependa kujifunza kutoka kwenye mawazo yako ya dhati na uzoefu. Hivyo, tafadhali usiogope kutoa majibu ya kweli.*

*Hakuna majibu yaliyo sahihi au yasio sahihi– Tunapenda zaidi kupata uzoefu wako binafsi na maoni yako. Mazungumzo yetu yatachukua wastani wa saa moja na nusu. Je, naweza pata ruhusa yako ya kushiriki kwenye mahojiano haya? (Kama ndio, endelea) Ninaomba pia ruhusa ya kurekodi majadiliano yetu kwa namna hii sitohitaji kuandika maelezo yako yote wakati tunazungumza. Je, una swali lolote kabla ya kuanza mjadala wa kikundi vya majadiliano?*

***Washa kinasa sauti.***

|  | **Questions** | **Objective** |
| --- | --- | --- |
| 1. | Tell me about yourself. *Ningependa kufahamu Zaidi kuhusu wewe.*   - - What is your training? *Je, umesomea nini?*   - What is your position in the facility? *Je, una nafasi gani hapa hospitalini? Majukumu yako ni yapi?*   - How long have you worked in this facility? *Umefanya kazi katika hospitali hii kwa muda gani? (miaka/miezi)* | Background and expectations |
| 2. | How were you informed about the PartoMa workshops? *Je, ulipataje taarifa kuhusu warsha (workshop)ya PartoMa?*   - - Who informed you about them? *Ni nani alikutaarifu?*   - What did they say? *Walikuambia nini?*   - Do you know how/why you were chosen to participate? *Je, unafahamu ni kwanini ulichaguliwa kushiriki?* |  |
| 3. | What were your expectations of the workshop? *Je, ulikua na matarajio gani kuhusu warsha/semina hii?*   - - What did you think it would be about? *Je, ulidhani warsha/semina hii ingekua inahusu nini?*   - What were you hoping to gain? *Je, ulitarajia kupata nini kutoka kwenye warsha/semina hii?* |  |
| 4. | I am interested to know your impressions of the workshop. What did you think about. *Ningependa kufahamu Zaidi kuhusu mtazamo wako juu ya warsha/semina ya PartoMa. Je, ulionaje yafuatayo:*   - The structure of the workshop from the beginning to the end. *Muundo wa warsha kuanzia mwanzo hadi mwisho.*   1. How did the workshop begin? *Je, warsha ilianzaje?*   2. What did you think about the flow of the activities? *Je, ulionaje mtiririko wawatukio yaliyotolewa?* - The language(s) of communication. *Lugha zilizotumika kwa ajili ya mawasiliano.* - The setting. *Eneo la workshop.* - Separate workshops for doctors, nurses and interns. *Utenganisho wa workshop kwa makundi i.e. madaktari, wauguzi, wanafunzi* - The presence of focal people who participated in facilitation. *Uwepo wa*    1. Did you think this was a good idea? *Je, uliona hili ni wazo zuri?*   2. Did you find them helpful or not? Why or why not? *Je, unahisi walikua na msaada wakati wa workshop? Iwapo ndio/hapana, kwanini?* - The lunch and transport arrangements. *Uwepo wa chakula cha asubuhi na posho kwa ajili ya usafiri.* - The time the workshop consumed i.e. 6-7 hours. *Muda uliotumika kwa ajili ya workshop.*   1. Was this convenient for you? *Je, muda uliopangwa ulikua sahihi/rahisi kwako?*   2. Did you have any other commitments on this day? *Je, ulikua na majukumu mengine yoyote kwa siku ile?*   3. Did you need to make any arrangements to be able to attend? *Je, ulihitaji kubadili mipango yako yoyote ili uweze kushiriki?* | Personal impressions of the workshop |
| 5. | I would like to know more in detail about your participation during the workshop particularly, going through and modifying the PartoMa booklet. *Ningependa kufahamu Zaidi kuhusu ushiriki wako wakati wa workshop haswa wakati wa kupitia na kufanya marekebisho kwenye kijitabu cha PartoMa.*   - Can you tell me about your experience of going through the booklet alone/in pairs/in a group? *Je, unaweza kuniambia kuhusu experience/uzoefu wako ya kupitia kijitabu cha PartoMa mwenyewe (binafsi) na kwenye group?* - How did you find the process of sharing feedback on the booklet with other participants and facilitators? *Je, ulionaje mchakato wa kutoa mrejesho kuhusu booklet/kijitabu cha PartoMa kwa washiriki wengine na wawezeshaji wa workshop?* - Can you give an example of how you took part in this process? E.g. What did you contribute in the process? *Je, unaweza kunipa mfano wa ni jinsi gani ulishiriki katika zoezi hili? Mf. Ulitoa mchango/mawazo gani kuhusu kitabu cha PartoMa?* - Can you share an example of one of the topics of discussion from the booklet that you feel were important during this process? What were some of the points raised? e.g. considering the context of the facility, cost of recommended medication. *Je, unaweza kunipa mfano wa mojawapo ya mada (topic) ambazo uliona zilikua za muhimu kutoka kwenye hii booklet? Je, ni mambo gani ya muhimu ambayo yalizungumziwa juu ya mada hii?* - Do you think that you were able to participate fully? *Je, unahisi kuwa uliweza kushiriki kikamilifu?*   - If yes, how so? If not, why not? What were some of the barriers/facilitators? *Iwapo ndio, kivipi? Iwapo hapana, kwanini? Unaweza kunitajia ni mambo yapi waliwezesha/kwamisha ushiriki wako katika workshop hii?* |  |
| 6. | ****For participants from the modification workshops****   - What did you think about the presentation and discussion of evidence shared during the workshop? E.g. oxytocin augmentation, trial of scar, RMC. *Je, ulionaje presentations/uwasilishi na mijadala juu ya evidence (ushahidi wa kisayansi) uliotolewa wakati wa workshop? Mf. Kuhusu matumizi ya oxytocin, trial of scar, respectful maternity care*    - Did the evidence make sense to you? If yes, what did you understand? If not, what was unclear? *Je, ushahidi huu ulikua unaeleweka kwako? Iwapo ndio, unaweza kunielezea ulielewa nini?Iwapo sio, ni nini ambacho hakikueleweka vizuri?* - How was your experience of making modifications to the booklet as a group (i.e. writing changes on the wall print outs)? How did you take part? Did you find this process useful? *Je, experience yako ya kufanya marekebisho kwenye booklet katika kikundi ilikuwaje? (i.e. kuandika maboresho yanayohitajika katika kurasa zilizobandikwa ukutani). Ulishiriki vipi kwenye zoezi hili? Je, zoezi hili lilikua na umuhimu wowote?* |  |
| 7. | One of the topics that was discussed was regarding the PartoMa workshops is regarding the set up and timing of the training workshops for the booklet. I would like to know your opinions about this. *Mojawapo ya mada zilizozungumzwa kwenye workshop ni kuhusu uwasilishi na muda wa semina za mafunzo yanayotokana na kitabi cha PartoMa. Ningependa kufahamu Zaidi kuhusu mtazamo wako juu ya hili.*   - When do you think would be the best time to have the workshops? *Je,unadhani ni muda upi ambao utafaa kutumika kwaajili ya kuendesha semina za PartoMa?* E.g. What do you think about having them after work hours like in Zanzibar? *Unaonaje kuhusu kuwa na semina baada ya muda wa kazi kama ilivyofanyika kule Zanzibar?* - Who (from your facility) do you think should facilitate these seminars?  *Ni nani ambae angefaa kuendesha hizi semina?* - Should any compensation be provided for attending these seminars? *Je, washiriki wa semina wanapaswa kupewa fidia yoyote kwa ajili ya kushiriki katika semina?* - What kind of compensation do you think would be best? *Ni fidia ya aina gani ambayo unadhani ingefaa zaidi?* - Who do you think should cover the cost for these seminars? *Je, ni nani ambae unadhani anatakiwa kugharimia semina hizi?* | Opinions regarding PartoMa seminars |
| 7. | What did you like most about the workshop? *Ni nini ambacho ulikipenda zaidi kuhusu workshop hii?*   - Going through the booklet, discussion and modification process, presentations/sharing of evidence, lunch and transport arrangements. *Kupitia kitabu cha PartoMa, majadiliano na zoezi la kufanya marekebisho, presentations/uwasilishi wa ushahidi wa kisayansi, chakula cha mchana na fidia ya usafiri.* - What made it enjoyable? *Ni nini ambacho kilikufurahisha kuhusu hili?* |  |
| 8. | What did you not like OR what do you think could have been improved? *Ni nini ambacho haukukipenda/kufurahia AU ni nini ambacho unahisi kingeweza kuboreshwa?* |  |
| 9. | Do you feel that the workshop met your expectations? How? *Je, unahisi workshop hii ilitimiza matarajio yako? Kivipi?* |  |
| 10. | I would like to know a bit more about your thoughts on the development of the clinical guidelines we use in our settings. *Ningependa kufahamu zaidi kuhusu mtazamo wako juu ya michakato ya kuunda miongozo ya utoaji huduma za afya (clinical guidelines) ambayo inatumika katika vituo vyetu vya afya hapa nchini.*   - How are our clinical guidelines usually developed? *Je, kwa ufahamu wako, unadhani miongozo ya utoaji huduma ya afya (clinical guidelines) huwa inatengenezwa vipi/kwa namna gani?* - Have you (or others in your work place) ever been involved in an effort to create guidelines? *Je, wewe (au wenzako katika eneo lako la kazi) mmewahi kuhusishwa katika mchakato wowote wa kutengeneza miongozo ya utoaji huduma ya afya?* - From your participation in this workshop, did you learn anything new about the development of clinical guidelines? If yes, what did you learn? *Kutokana na ushiriki wako katika workshop hii, je umejifunza chochote kipya juu ya uundaji wa miongozo ya utoaji huduma ya afya? Umejifunza nini?*   - For example, what do you think of the role of evidence in the creation of clinical guidelines? *Kwa mfano, unadhani ushahidi wa kisayansi una nafasi gani katika kuunda miongozo ya utoaji huduma ya afya?* | Knowledge and perceptions on the development of guidelines |
|  | **Closing the interview**  **Thank you for your time and for agreeing to take part in this interview. Before we close the interview. Hayo ndio maswali yangu. *Asante sana kwa kukubali kushiriki na kutumia muda wako kuzungumza na mimi. Kabla hatujafunga;***   1. Do you have any further comments/anything else that you would like to share about your participation during the PartoMa workshop? *Je, una nyongeza yoyote au chochote cha ziada ambacho ungependa kuzungumzia juu ya ushiriki wako katika workshop ya/za PartoMa?* 2. Do you have any questions about the study that you would like to ask? *Je una maswali yoyote juu ya utafiti wetu?*   **Asante sana.** |  |
